# (*E*)-2-[4-(2-Chloro­phen­yl)but-3-en-2-yl­idene]malononitrile

**DOI:** 10.1107/S1600536811037159

**Published:** 2011-09-17

**Authors:** Tai-Ran Kang

**Affiliations:** a College of Chemistry and Chemical Engineering, China West Normal University, Nanchong 637002, People’s Republic of China

## Abstract

There are two independent but virtually identical mol­ecules in the asymmetric unit of the title compound, C_13_H_19_ClN_2_. Each mol­ecular skeleton displays an approximately planar structure except for the methyl group [the r.m.s. deviations for all 16 non-H atoms are 0.039 (mol­ecule 1) and 0.056 Å (mol­ecule 2)]. An *E* configuration is found about each of the C=C bonds. The crystal packing is stabilized by C—H⋯N inter­actions that connect the independent mol­ecules into supra­molecular chains along the *c*-axis direction.

## Related literature

For the use of malononitrile-containing compounds as building blocks in synthesis, see: Liu *et al.* (2002 ▶); Sepiol & Milart (1985 ▶); Zhang *et al.* (2003 ▶). For a related structure, see: Kang & Chen (2009 ▶).
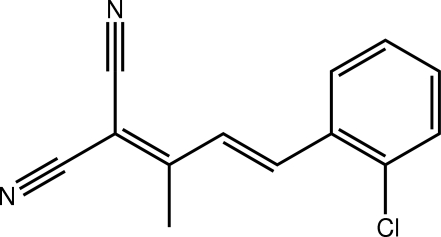


## Experimental

### 

#### Crystal data

C_13_H_9_ClN_2_
*M* *_r_* = 228.67
Triclinic, 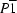

*a* = 7.7177 (2) Å
*b* = 11.0539 (5) Å
*c* = 14.7236 (5) Å
α = 91.260 (3)°
β = 103.992 (3)°
γ = 106.357 (3)°
*V* = 1163.99 (7) Å^3^
*Z* = 4
Cu *K*α radiation
μ = 2.67 mm^−1^
*T* = 291 K
0.35 × 0.32 × 0.30 mm

#### Data collection

Oxford Diffraction Xcalibur Sapphire3 Gemini ultra diffractometer
Absorption correction: multi-scan (*CrysAlis PRO*; Oxford Diffraction, 2009 ▶) *T*_min_ = 0.455, *T*_max_ = 0.502
9739 measured reflections
4135 independent reflections
3770 reflections with *I* > 2σ(*I*)
*R*_int_ = 0.027

#### Refinement

*R*[*F* ^2^ > 2σ(*F* ^2^)] = 0.048
*wR*(*F* ^2^) = 0.140
*S* = 1.04
4135 reflections
291 parameters
H-atom parameters constrained
Δρ_max_ = 0.45 e Å^−3^
Δρ_min_ = −0.20 e Å^−3^

### 

Data collection: *CrysAlis PRO* (Oxford Diffraction, 2009 ▶); cell refinement: *CrysAlis PRO*; data reduction: *CrysAlis PRO*; program(s) used to solve structure: *SHELXS97* (Sheldrick, 2008 ▶); program(s) used to refine structure: *SHELXL97* (Sheldrick, 2008 ▶); molecular graphics: *ORTEP-3* (Farrugia, 1997 ▶); software used to prepare material for publication: *SHELXL97*.

## Supplementary Material

Crystal structure: contains datablock(s) global, I. DOI: 10.1107/S1600536811037159/tk2789sup1.cif

Structure factors: contains datablock(s) I. DOI: 10.1107/S1600536811037159/tk2789Isup2.hkl

Supplementary material file. DOI: 10.1107/S1600536811037159/tk2789Isup3.cml

Additional supplementary materials:  crystallographic information; 3D view; checkCIF report

## Figures and Tables

**Table 1:** Hydrogen-bond geometry (Å, °)

*D*—H⋯*A*	*D*—H	H⋯*A*	*D*⋯*A*	*D*—H⋯*A*
C10—H10*C*⋯N3^i^	0.96	2.62	3.564 (3)	166 (1)

Symmetry code: (i) 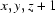.

**Fig. 1.**
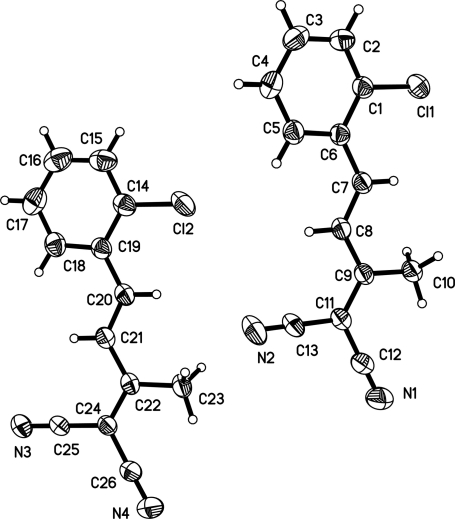
The molecular structure of (I) showing atom labelling scheme and 30% probability displacement ellipsoids (arbitrary spheres for H atoms).

## supplementary crystallographic information

### Comment

The chemistry of ylidene malononitrile has been studied extensively for ring closure reactions, with compounds containing newly formed five- or six-membered rings, such as indanes (Zhang *et al.*, 2003), naphthalenes (Liu *et al.*, 2002) and benzenes (Sepiol & Milart, 1985) being obtained. Some crystal structures involving ylidene malononitrile groups have been published, including a recent report from our laboratory (Kang & Chen, 2009). As a part of our interest in the synthesis of some complex ring systems, we investigated the title compound (I), which is a diene reagent in the Diels-Alder reaction. We report herein the crystal structure of (I).

Two independent molecules comprise the asymmetric unit of (I), Fig. 1. The molecular skeleton displays an approximately planar arrangement in each case. The chlorobenzene ring and 2-propylidenemalononitrile groups are located on opposite sides of the double bond to which they are attached, showing an *E* configuration. The crystal packing is stabilized by C—H···N interactions (Table 1).

### Experimental

2-(Propan-2-ylidene)malononitrile (0.212 g, 2 mmol) and 2-chlorobenzaldehyde (0.28 g, 2 mmol) were dissolved in 2-propanol (2 ml). To the solution was added piperidine (0.017 g, 0.2 mmol). The solution was then stirred for 24 h at 343 K. The reaction mixture was cooled to room temperature and the solution was filtered to obtain a white solid. Recrystallization from hot ethanol afforded the pure compound. Single crystals of (I) were obtained by slow evaporation of its ethyl acetate solution.

### Refinement

The carbon-bound hydrogen atoms were placed in calculated positions, with C—H = 0.93–0.96 Å, and refined using a riding model, with *U*_iso_(H) =1.5*U*_eq_(C) for methyl H atoms and *U*_iso_(H) =1.2*U*_eq_(C) for the others.

### Crystal data

**Table:**

C_13_H_9_ClN_2_	*Z* = 4
*M**_r_* = 228.67	*F*(000) = 472
Triclinic, *P*1	*D*_x_ = 1.305 Mg m^−^^3^
Hall symbol: -P 1	Cu *K*α radiation, λ = 1.54184 Å
*a* = 7.7177 (2) Å	Cell parameters from 6315 reflections
*b* = 11.0539 (5) Å	θ = 3.1–72.1°
*c* = 14.7236 (5) Å	µ = 2.67 mm^−^^1^
α = 91.260 (3)°	*T* = 291 K
β = 103.992 (3)°	Block, yellow
γ = 106.357 (3)°	0.35 × 0.32 × 0.30 mm
*V* = 1163.99 (7) Å^3^	

### Data collection

**Table:**

Oxford Diffraction Xcalibur Sapphire3 Gemini ultra diffractometer	4135 independent reflections
Radiation source: Enhance Ultra (Cu) X-ray Source	3770 reflections with *I* > 2σ(*I*)
mirror	*R*_int_ = 0.027
Detector resolution: 15.9149 pixels mm^-1^	θ_max_ = 67.1°, θ_min_ = 3.1°
ω scans	*h* = −9→7
Absorption correction: multi-scan (*CrysAlis PRO*; Oxford Diffraction, 2009)	*k* = −13→13
*T*_min_ = 0.455, *T*_max_ = 0.502	*l* = −17→17
9739 measured reflections	

### Refinement

**Table:**

Refinement on *F*^2^	Primary atom site location: structure-invariant direct methods
Least-squares matrix: full	Secondary atom site location: difference Fourier map
*R*[*F*^2^ > 2σ(*F*^2^)] = 0.048	Hydrogen site location: inferred from neighbouring sites
*wR*(*F*^2^) = 0.140	H-atom parameters constrained
*S* = 1.04	*w* = 1/[σ^2^(*F*_o_^2^) + (0.0727*P*)^2^ + 0.290*P*] where *P* = (*F*_o_^2^ + 2*F*_c_^2^)/3
4135 reflections	(Δ/σ)_max_ = 0.001
291 parameters	Δρ_max_ = 0.45 e Å^−^^3^
0 restraints	Δρ_min_ = −0.20 e Å^−^^3^

### Special details

**Table:**

Geometry. All e.s.d.'s (except the e.s.d. in the dihedral angle between two l.s. planes) are estimated using the full covariance matrix. The cell e.s.d.'s are taken into account individually in the estimation of e.s.d.'s in distances, angles and torsion angles; correlations between e.s.d.'s in cell parameters are only used when they are defined by crystal symmetry. An approximate (isotropic) treatment of cell e.s.d.'s is used for estimating e.s.d.'s involving l.s. planes.
Refinement. Refinement of *F*^2^ against ALL reflections. The weighted *R*-factor *wR* and goodness of fit *S* are based on *F*^2^, conventional *R*-factors *R* are based on *F*, with *F* set to zero for negative *F*^2^. The threshold expression of *F*^2^ > σ(*F*^2^) is used only for calculating *R*-factors(gt) *etc*. and is not relevant to the choice of reflections for refinement. *R*-factors based on *F*^2^ are statistically about twice as large as those based on *F*, and *R*- factors based on ALL data will be even larger.

### Fractional atomic coordinates and isotropic or equivalent isotropic displacement parameters (Å^2^)

**Table:**

	*x*	*y*	*z*	*U*_iso_*/*U*_eq_	
C24	0.5347 (2)	0.25777 (17)	0.36952 (12)	0.0541 (4)	
C9	0.6600 (2)	0.30400 (18)	0.94779 (12)	0.0536 (4)	
C8	0.6947 (3)	0.43885 (18)	0.96046 (13)	0.0562 (4)	
H8	0.6495	0.4786	0.9088	0.067*	
C1	0.9378 (3)	0.71648 (18)	1.14140 (13)	0.0569 (4)	
C23	0.5469 (3)	0.2649 (2)	0.53865 (14)	0.0670 (5)	
H23A	0.4703	0.1791	0.5194	0.100*	
H23C	0.4796	0.3106	0.5656	0.100*	
H23B	0.6592	0.2654	0.5846	0.100*	
C22	0.5964 (3)	0.32662 (18)	0.45502 (13)	0.0551 (4)	
N1	0.4827 (4)	−0.0031 (2)	0.83682 (16)	0.0942 (7)	
C11	0.5561 (3)	0.23861 (18)	0.86407 (13)	0.0574 (4)	
C19	0.8945 (3)	0.66283 (18)	0.55896 (14)	0.0588 (5)	
C6	0.8253 (2)	0.64747 (17)	1.05665 (12)	0.0524 (4)	
C26	0.4247 (3)	0.1281 (2)	0.35739 (15)	0.0697 (5)	
C12	0.5155 (3)	0.1042 (2)	0.84862 (15)	0.0679 (5)	
C25	0.5774 (3)	0.30988 (19)	0.28661 (13)	0.0600 (5)	
C14	0.9546 (3)	0.7332 (2)	0.64619 (15)	0.0651 (5)	
C5	0.7504 (3)	0.71784 (19)	0.98811 (14)	0.0613 (5)	
H5	0.6738	0.6759	0.9307	0.074*	
C4	0.7863 (3)	0.8465 (2)	1.00278 (17)	0.0716 (6)	
H4	0.7347	0.8903	0.9557	0.086*	
N2	0.4120 (3)	0.3468 (2)	0.72447 (14)	0.0907 (6)	
C10	0.7382 (3)	0.2352 (2)	1.02658 (14)	0.0645 (5)	
H10A	0.6969	0.1460	1.0073	0.097*	
H10C	0.6955	0.2506	1.0806	0.097*	
H10B	0.8723	0.2650	1.0423	0.097*	
C20	0.7793 (3)	0.53241 (19)	0.54577 (13)	0.0594 (5)	
H20	0.7489	0.4972	0.5987	0.071*	
C21	0.7120 (3)	0.45749 (18)	0.46499 (13)	0.0577 (4)	
H21	0.7412	0.4915	0.4114	0.069*	
C7	0.7870 (2)	0.51104 (18)	1.04108 (13)	0.0544 (4)	
H7	0.8312	0.4701	1.0922	0.065*	
C18	0.9567 (3)	0.7248 (2)	0.48501 (16)	0.0703 (5)	
H18	0.9197	0.6815	0.4254	0.084*	
C17	1.0704 (3)	0.8474 (2)	0.4987 (2)	0.0855 (7)	
H17	1.1080	0.8863	0.4484	0.103*	
C2	0.9749 (3)	0.8458 (2)	1.15697 (17)	0.0730 (6)	
H2	1.0507	0.8888	1.2142	0.088*	
C13	0.4755 (3)	0.2988 (2)	0.78642 (14)	0.0661 (5)	
C15	1.0718 (3)	0.8564 (2)	0.6601 (2)	0.0844 (7)	
H15	1.1119	0.9008	0.7194	0.101*	
C3	0.8988 (3)	0.9107 (2)	1.08720 (19)	0.0776 (6)	
H3	0.9233	0.9980	1.0971	0.093*	
C16	1.1285 (4)	0.9126 (3)	0.5857 (3)	0.0941 (8)	
H16	1.2068	0.9954	0.5947	0.113*	
N3	0.6097 (3)	0.3519 (2)	0.22018 (13)	0.0802 (5)	
N4	0.3349 (4)	0.0253 (2)	0.34942 (17)	0.1014 (8)	
Cl1	1.03787 (8)	0.63948 (6)	1.23196 (4)	0.0778 (2)	
Cl2	0.88209 (9)	0.66910 (6)	0.74273 (4)	0.0836 (2)	

### Atomic displacement parameters (Å^2^)

**Table:**

	*U*^11^	*U*^22^	*U*^33^	*U*^12^	*U*^13^	*U*^23^
C24	0.0527 (9)	0.0603 (10)	0.0449 (9)	0.0145 (8)	0.0070 (7)	0.0042 (8)
C9	0.0564 (10)	0.0620 (10)	0.0417 (9)	0.0157 (8)	0.0137 (8)	0.0040 (7)
C8	0.0603 (10)	0.0620 (11)	0.0433 (9)	0.0149 (8)	0.0113 (8)	0.0084 (8)
C1	0.0525 (9)	0.0617 (11)	0.0491 (10)	0.0086 (8)	0.0089 (8)	0.0051 (8)
C23	0.0747 (12)	0.0774 (13)	0.0473 (10)	0.0197 (10)	0.0156 (9)	0.0124 (9)
C22	0.0581 (10)	0.0639 (11)	0.0451 (9)	0.0240 (8)	0.0093 (8)	0.0088 (8)
N1	0.1175 (17)	0.0700 (13)	0.0813 (14)	0.0206 (12)	0.0100 (12)	−0.0096 (11)
C11	0.0626 (10)	0.0653 (11)	0.0435 (9)	0.0187 (9)	0.0129 (8)	0.0020 (8)
C19	0.0597 (10)	0.0621 (11)	0.0544 (11)	0.0267 (9)	0.0044 (8)	0.0055 (8)
C6	0.0483 (9)	0.0575 (10)	0.0474 (9)	0.0100 (7)	0.0115 (7)	0.0075 (7)
C26	0.0694 (12)	0.0719 (14)	0.0546 (11)	0.0062 (10)	0.0094 (10)	0.0008 (9)
C12	0.0750 (13)	0.0696 (13)	0.0526 (11)	0.0178 (10)	0.0094 (10)	−0.0039 (9)
C25	0.0649 (11)	0.0634 (11)	0.0440 (10)	0.0142 (9)	0.0062 (8)	−0.0008 (8)
C14	0.0636 (11)	0.0714 (12)	0.0598 (11)	0.0322 (10)	0.0011 (9)	0.0005 (9)
C5	0.0567 (10)	0.0682 (12)	0.0538 (11)	0.0139 (9)	0.0093 (8)	0.0133 (9)
C4	0.0713 (13)	0.0674 (13)	0.0790 (15)	0.0220 (10)	0.0216 (11)	0.0246 (11)
N2	0.1135 (16)	0.1141 (17)	0.0487 (11)	0.0514 (14)	0.0078 (10)	0.0067 (10)
C10	0.0774 (13)	0.0643 (12)	0.0482 (10)	0.0223 (10)	0.0082 (9)	0.0066 (9)
C20	0.0618 (11)	0.0675 (11)	0.0475 (10)	0.0225 (9)	0.0074 (8)	0.0068 (8)
C21	0.0598 (10)	0.0646 (11)	0.0473 (10)	0.0191 (9)	0.0102 (8)	0.0077 (8)
C7	0.0527 (9)	0.0619 (11)	0.0448 (9)	0.0133 (8)	0.0094 (7)	0.0065 (8)
C18	0.0731 (13)	0.0788 (14)	0.0644 (13)	0.0338 (11)	0.0136 (10)	0.0138 (10)
C17	0.0732 (14)	0.0788 (16)	0.110 (2)	0.0272 (12)	0.0273 (14)	0.0291 (15)
C2	0.0726 (13)	0.0618 (12)	0.0689 (13)	0.0011 (10)	0.0125 (11)	−0.0047 (10)
C13	0.0773 (13)	0.0785 (13)	0.0411 (10)	0.0257 (11)	0.0104 (9)	−0.0009 (9)
C15	0.0735 (14)	0.0753 (15)	0.0921 (18)	0.0239 (12)	−0.0022 (13)	−0.0113 (13)
C3	0.0828 (15)	0.0555 (12)	0.0920 (17)	0.0122 (10)	0.0276 (13)	0.0094 (11)
C16	0.0732 (15)	0.0717 (15)	0.127 (3)	0.0179 (12)	0.0117 (16)	0.0060 (16)
N3	0.0992 (14)	0.0858 (13)	0.0485 (10)	0.0164 (11)	0.0189 (9)	0.0050 (9)
N4	0.1103 (17)	0.0789 (14)	0.0845 (15)	−0.0136 (13)	0.0191 (13)	−0.0043 (11)
Cl1	0.0806 (4)	0.0843 (4)	0.0513 (3)	0.0177 (3)	−0.0067 (2)	0.0067 (2)
Cl2	0.0973 (4)	0.1005 (5)	0.0477 (3)	0.0347 (3)	0.0035 (3)	−0.0035 (3)

### Geometric parameters (Å, °)

**Table:**

C24—C22	1.362 (3)	C25—N3	1.144 (3)
C24—C26	1.427 (3)	C14—C15	1.386 (3)
C24—C25	1.432 (3)	C14—Cl2	1.744 (2)
C9—C11	1.357 (3)	C5—C4	1.372 (3)
C9—C8	1.438 (3)	C5—H5	0.9300
C9—C10	1.499 (3)	C4—C3	1.377 (3)
C8—C7	1.335 (3)	C4—H4	0.9300
C8—H8	0.9300	N2—C13	1.143 (3)
C1—C2	1.379 (3)	C10—H10A	0.9600
C1—C6	1.398 (3)	C10—H10C	0.9600
C1—Cl1	1.738 (2)	C10—H10B	0.9600
C23—C22	1.498 (3)	C20—C21	1.336 (3)
C23—H23A	0.9600	C20—H20	0.9300
C23—H23C	0.9600	C21—H21	0.9300
C23—H23B	0.9600	C7—H7	0.9300
C22—C21	1.453 (3)	C18—C17	1.370 (3)
N1—C12	1.141 (3)	C18—H18	0.9300
C11—C12	1.430 (3)	C17—C16	1.365 (4)
C11—C13	1.435 (3)	C17—H17	0.9300
C19—C14	1.391 (3)	C2—C3	1.376 (3)
C19—C18	1.409 (3)	C2—H2	0.9300
C19—C20	1.445 (3)	C15—C16	1.372 (4)
C6—C5	1.401 (3)	C15—H15	0.9300
C6—C7	1.454 (3)	C3—H3	0.9300
C26—N4	1.139 (3)	C16—H16	0.9300
			
C22—C24—C26	121.80 (17)	C6—C5—H5	118.9
C22—C24—C25	122.40 (17)	C5—C4—C3	120.0 (2)
C26—C24—C25	115.80 (17)	C5—C4—H4	120.0
C11—C9—C8	119.85 (17)	C3—C4—H4	120.0
C11—C9—C10	119.80 (18)	C9—C10—H10A	109.5
C8—C9—C10	120.35 (16)	C9—C10—H10C	109.5
C7—C8—C9	124.63 (17)	H10A—C10—H10C	109.5
C7—C8—H8	117.7	C9—C10—H10B	109.5
C9—C8—H8	117.7	H10A—C10—H10B	109.5
C2—C1—C6	122.53 (19)	H10C—C10—H10B	109.5
C2—C1—Cl1	117.46 (16)	C21—C20—C19	126.43 (19)
C6—C1—Cl1	120.01 (15)	C21—C20—H20	116.8
C22—C23—H23A	109.5	C19—C20—H20	116.8
C22—C23—H23C	109.5	C20—C21—C22	124.53 (18)
H23A—C23—H23C	109.5	C20—C21—H21	117.7
C22—C23—H23B	109.5	C22—C21—H21	117.7
H23A—C23—H23B	109.5	C8—C7—C6	126.29 (17)
H23C—C23—H23B	109.5	C8—C7—H7	116.9
C24—C22—C21	120.17 (17)	C6—C7—H7	116.9
C24—C22—C23	119.18 (18)	C17—C18—C19	121.7 (2)
C21—C22—C23	120.64 (17)	C17—C18—H18	119.1
C9—C11—C12	121.86 (18)	C19—C18—H18	119.1
C9—C11—C13	122.42 (18)	C16—C17—C18	120.3 (3)
C12—C11—C13	115.70 (18)	C16—C17—H17	119.9
C14—C19—C18	116.2 (2)	C18—C17—H17	119.9
C14—C19—C20	121.75 (19)	C3—C2—C1	119.5 (2)
C18—C19—C20	122.03 (19)	C3—C2—H2	120.3
C1—C6—C5	115.78 (18)	C1—C2—H2	120.3
C1—C6—C7	122.04 (17)	N2—C13—C11	179.7 (2)
C5—C6—C7	122.18 (17)	C16—C15—C14	119.6 (3)
N4—C26—C24	178.2 (3)	C16—C15—H15	120.2
N1—C12—C11	179.5 (3)	C14—C15—H15	120.2
N3—C25—C24	179.2 (2)	C2—C3—C4	120.0 (2)
C15—C14—C19	121.9 (2)	C2—C3—H3	120.0
C15—C14—Cl2	117.28 (19)	C4—C3—H3	120.0
C19—C14—Cl2	120.77 (17)	C17—C16—C15	120.3 (2)
C4—C5—C6	122.21 (19)	C17—C16—H16	119.9
C4—C5—H5	118.9	C15—C16—H16	119.9
			
C11—C9—C8—C7	176.58 (19)	C1—C6—C5—C4	0.6 (3)
C10—C9—C8—C7	−3.4 (3)	C7—C6—C5—C4	179.95 (18)
C26—C24—C22—C21	178.43 (18)	C6—C5—C4—C3	−0.3 (3)
C25—C24—C22—C21	−0.8 (3)	C14—C19—C20—C21	178.88 (19)
C26—C24—C22—C23	−0.3 (3)	C18—C19—C20—C21	−2.9 (3)
C25—C24—C22—C23	−179.49 (18)	C19—C20—C21—C22	−179.99 (17)
C8—C9—C11—C12	−179.02 (18)	C24—C22—C21—C20	−179.91 (19)
C10—C9—C11—C12	1.0 (3)	C23—C22—C21—C20	−1.2 (3)
C8—C9—C11—C13	−0.8 (3)	C9—C8—C7—C6	179.82 (17)
C10—C9—C11—C13	179.17 (19)	C1—C6—C7—C8	−174.14 (19)
C2—C1—C6—C5	−0.6 (3)	C5—C6—C7—C8	6.5 (3)
Cl1—C1—C6—C5	179.76 (14)	C14—C19—C18—C17	0.2 (3)
C2—C1—C6—C7	−179.94 (18)	C20—C19—C18—C17	−178.10 (19)
Cl1—C1—C6—C7	0.4 (3)	C19—C18—C17—C16	0.8 (3)
C22—C24—C26—N4	35 (9)	C6—C1—C2—C3	0.2 (3)
C25—C24—C26—N4	−146 (9)	Cl1—C1—C2—C3	179.92 (18)
C9—C11—C12—N1	56 (43)	C9—C11—C13—N2	85 (47)
C13—C11—C12—N1	−122 (43)	C12—C11—C13—N2	−96 (47)
C22—C24—C25—N3	−90 (17)	C19—C14—C15—C16	1.2 (3)
C26—C24—C25—N3	91 (17)	Cl2—C14—C15—C16	−178.09 (19)
C18—C19—C14—C15	−1.2 (3)	C1—C2—C3—C4	0.1 (4)
C20—C19—C14—C15	177.10 (19)	C5—C4—C3—C2	−0.1 (4)
C18—C19—C14—Cl2	178.12 (14)	C18—C17—C16—C15	−0.8 (4)
C20—C19—C14—Cl2	−3.6 (3)	C14—C15—C16—C17	−0.2 (4)

### Hydrogen-bond geometry (Å, °)

**Table:**

*D*—H···*A*	*D*—H	H···*A*	*D*···*A*	*D*—H···*A*
C10—H10C···N3^i^	0.96	2.62	3.564 (3)	166.(1)

Symmetry codes: (i) *x*, *y*, *z*+1.
